# A Conservative and Multidisciplinary Approach to Boerhaave Syndrome: A Case Report

**DOI:** 10.7759/cureus.59602

**Published:** 2024-05-03

**Authors:** Ricardo Ribeiro, Paulo Cardoso, Florissandra Santos

**Affiliations:** 1 General Surgery, Centro Hospitalar Universitário do Algarve, Faro, PRT; 2 Surgery, Centro Hospitalar Universitário do Algarve, Faro, PRT

**Keywords:** endoscopy, emergency, spontaneous esophageal rupture, conservative treatment, boerhaave’s syndrome

## Abstract

Boerhaave’s syndrome is a life-threatening spontaneous esophageal rupture, usually in its distal part. It generally develops after situations that suddenly increase the intraesophageal pressure, such as, during or after persistent vomiting. Despite it being a rare condition in clinical practice, it has a high mortality rate (18-39%). Treatment can be approached conservatively, endoscopically, or surgically. The more invasive the treatment, the worse the prognosis.

This paper presents a healthy 62-year-old man who resorted to the emergency department complaining of lower back and left scapular pain, after two non-bilious episodes of vomiting. There was no history of any trauma, vigorous physical exercise or previous similar episodes. He was alert, hemodynamically stable, and without any airway compromise or respiratory distress. At the physical exam, non-painful subcutaneous crepitations were palpable in the left cervical region without palpable masses. Chest examination finds reduced air entry on the left pulmonary base. Hence, Boerhaave’s syndrome was suspected. CT scan revealed a pneumomediastinum and a left pulmonary collection. Oxygen therapy, blood cultures, empirical antibiotic therapy, and thoracic tube drainage were performed. The upper digestive endoscopy revealed the perforation in the distal esophagus, and an over-the-scope clip, a covered endoprosthesis and nasojejunal tube feeding were placed.

The patient was admitted to the Intermediate Care Unit for stabilization. He was discharged home on the 33^rd^ day and remains well at two months of follow-up.

Delayed diagnosis and treatment are the principal causes of high mortality in Boerhaave’s syndrome. There is no standard treatment option. In this case report, given the patient’s stable clinical condition, the authors used a non-surgical conservative treatment, allowing for a delayed esophageal repair.

## Introduction

Boerhaave's syndrome consists of spontaneous longitudinal transmural rupture of the esophagus. It is an extremely rare condition [1] with a high mortality rate (18-39%) [2,3]. It is caused by situations that rapidly increase intraesophageal pressure [1,4].

The clinical presentation of spontaneous esophageal rupture varies depending on the location of the rupture. In 50% of cases, it presents as Mackler's triad: vomiting, lower thoracic pain, and subcutaneous emphysema.

If the diagnosis is not established promptly and appropriate therapeutic measures are not taken, serious complications may ensue, potentially resulting in a poor prognosis [1,4].

Treatment can be approached conservatively, endoscopically, or surgically, with a worse prognosis associated with more invasive interventions [1].

The authors present a case of Boerhaave's syndrome treated in a multidisciplinary and non-surgical approach.

## Case presentation

An otherwise healthy 62-year-old Caucasian man presented to our Emergency Department complaining of lower back and left scapular pain for the previous 24 hours, after two non-bilious episodes of vomiting. After 3 hours, the pain worsened, extending to the left hemithorax. The pain was constant, tight, of moderate intensity and with pleuritic characteristics. The pain did not improve with the position, or with simple analgesia. He denied constitutional symptoms (e.g., weight loss, anorexia and fever), dyspnea, productive cough, dizziness, lipothymia, chills and sweating. There was no history of any trauma, vigorous physical exercise or previous similar episodes. He did not take any regular medication and has no allergies. He smokes two cigarettes a day for 40 years.

On examination, he was alert and responsive without any airway compromise or respiratory distress. He had a respiratory rate of 19 breaths per minute with an oxygen saturation of 94% on room air. His pulse rate was 71 beats per minute with a blood pressure of 151/76 mmHg. He was apyretic. Non-painful subcutaneous crepitations were palpable in the left cervical region without palpable masses. Chest examination finds reduced air entry on the left pulmonary base. The rest of his physical examination was unremarkable.

**Figure 1:**
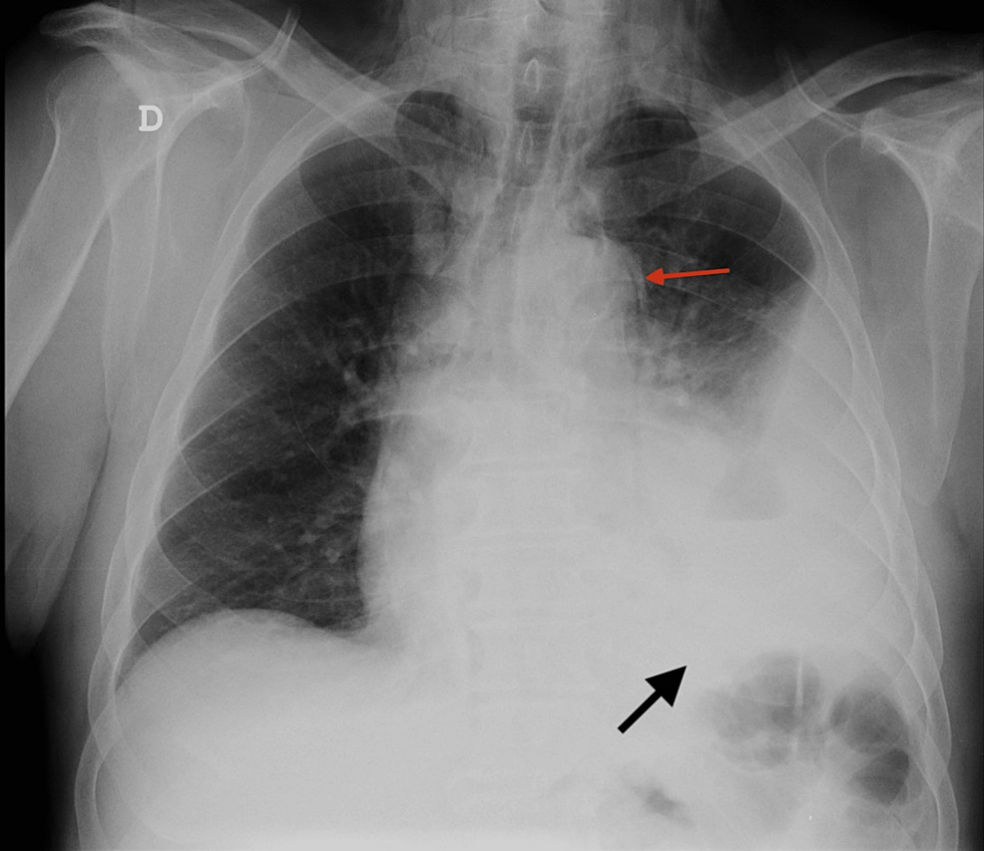
Chest X-ray revealing a pneumomediastinum (red arrow) and a left pleural effusion (black arrow).

The arterial blood gases revealed type 1 respiratory failure (PaO2 58 mmHg, PaCO2 37 mmHg, SpO2 90.6%) and the baseline blood tests revealed leukocytosis with neutrophilia and C-reactive protein of 142 mg/dL. An electrocardiogram showed a normal sinus rhythm. A plain posterior-anterior chest radiograph revealed pneumomediastinum and left pleural effusion (Figure 1).

**Figure 2:**
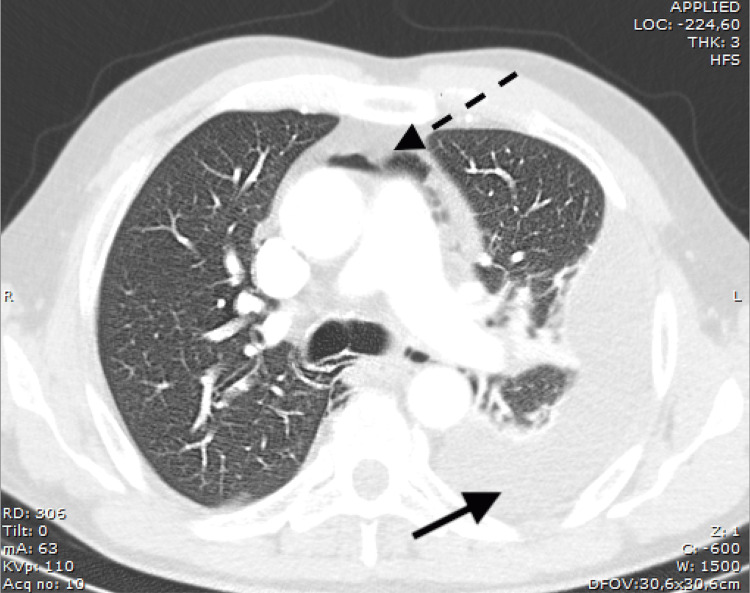
Axial CT acquisitions revealing a pneumomediastinum (dashed arrow) and a left pulmonary collection (bold arrow).

An axial computed tomography of his chest was performed and confirmed the radiographic findings: a pneumomediastinum (dashed arrow) and a left pulmonary collection (bold arrow) (Figure 2).

**Figure 3:**
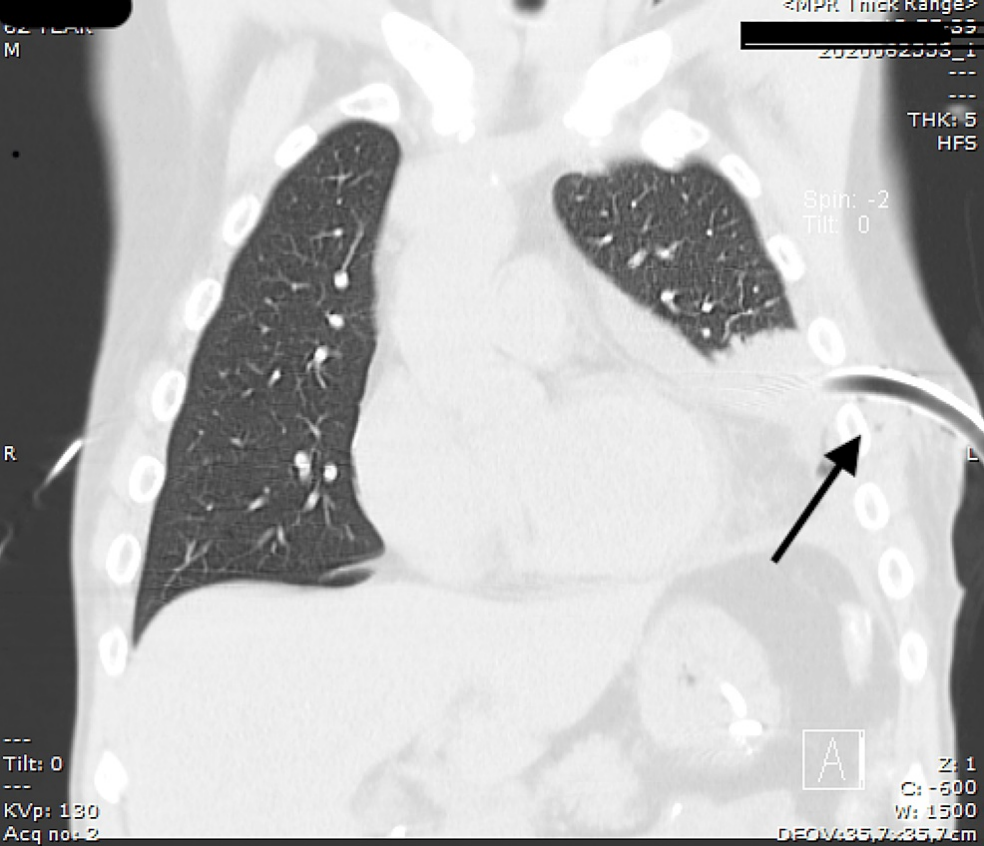
Coronal CT acquisitions showing the position of the first chest tube, placed in the fifth intercostal space (black arrow).

He underwent oxygen therapy aiming for a target oxygen saturation of 94%, blood cultures were obtained, and empirical antibiotic therapy with Ceftriaxone and Clindamycin was initiated. Chest drainage was performed (Figure 3).

The fluid quantitative cytological study showed a leukocytosis (19.935 cells/mm^3^), proteins of 4 g/dL, lactate dehydrogenase of 1.444 U/L and amylase of 518 U/L.

**Figure 4:**
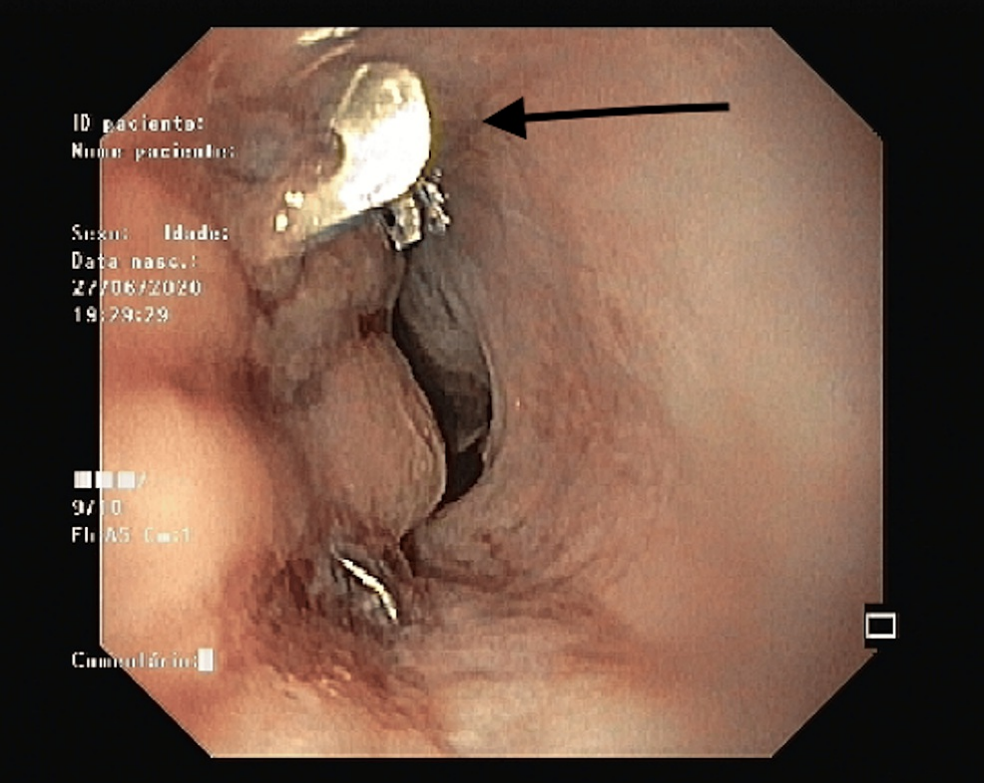
Upper digestive endoscopy showing the clip (black arrow) in the distal third of the esophagus.

**Figure 5:**
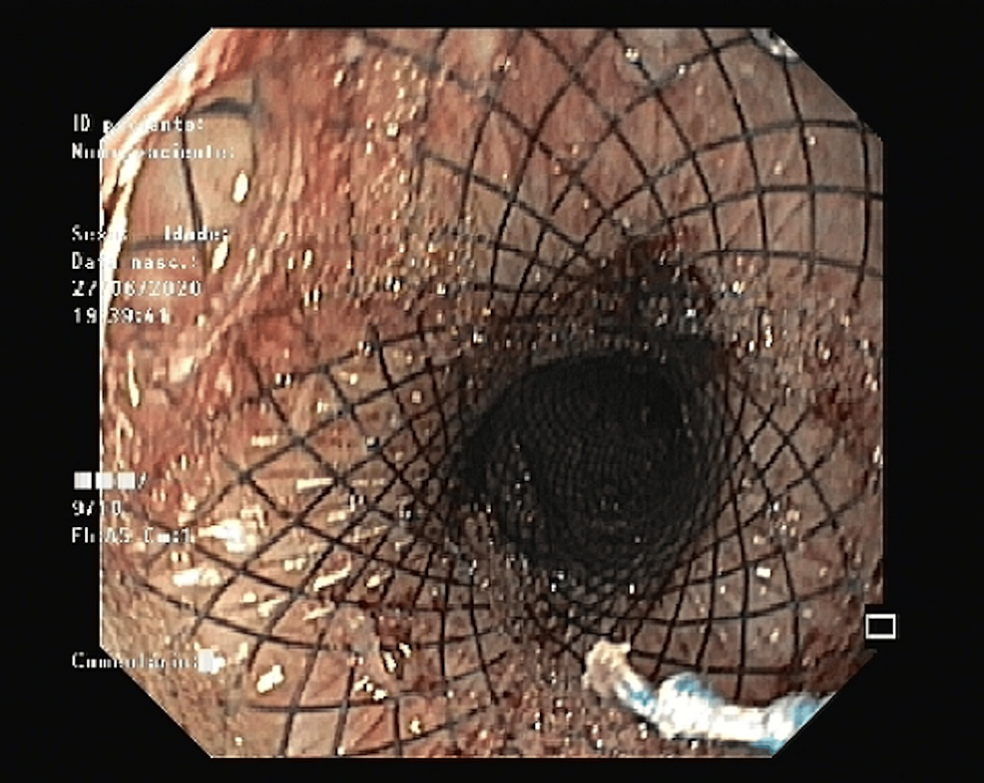
Endoscopy showing the metal prosthesis plastic-covered.

An upper digestive endoscopy (UDE) was also performed, and the images showed a punctual perforation hole in the distal third of the esophagus. The patient underwent the placement of an OTSC clip (over-the-scope clip), metallic-covered endoprosthesis and nasojejunal (NJ) tube feeding (Figures 4, 5).

The patient was admitted to the Intermediate Care Unit (IMCU) after discussing the clinical case with the following specialties: General Surgery, Pneumology, Gastroenterology, Internal Medicine, Intensive Care Medicine and Radiology. The Cardiothoracic Surgery of another reference hospital was also contacted, having validated the instituted therapy and having proposed a conservative management.

The hospital stay lasted 33 days, nine of which in IMCU and 24 in the surgical ward. During hospitalization, four asynchronous chest drains were placed to drain the different empyemas and obtain material for culture. The metal prosthesis was removed on the 17th day of hospitalization.

The patient remained hemodynamically stable during the hospital stay. He was discharged on the 33rd day, given the clinical improvement. Antibiotic therapy had been suspended for the previous six days to the discharge date, and the chest tube had a small amount of serous content.

**Figure 6:**
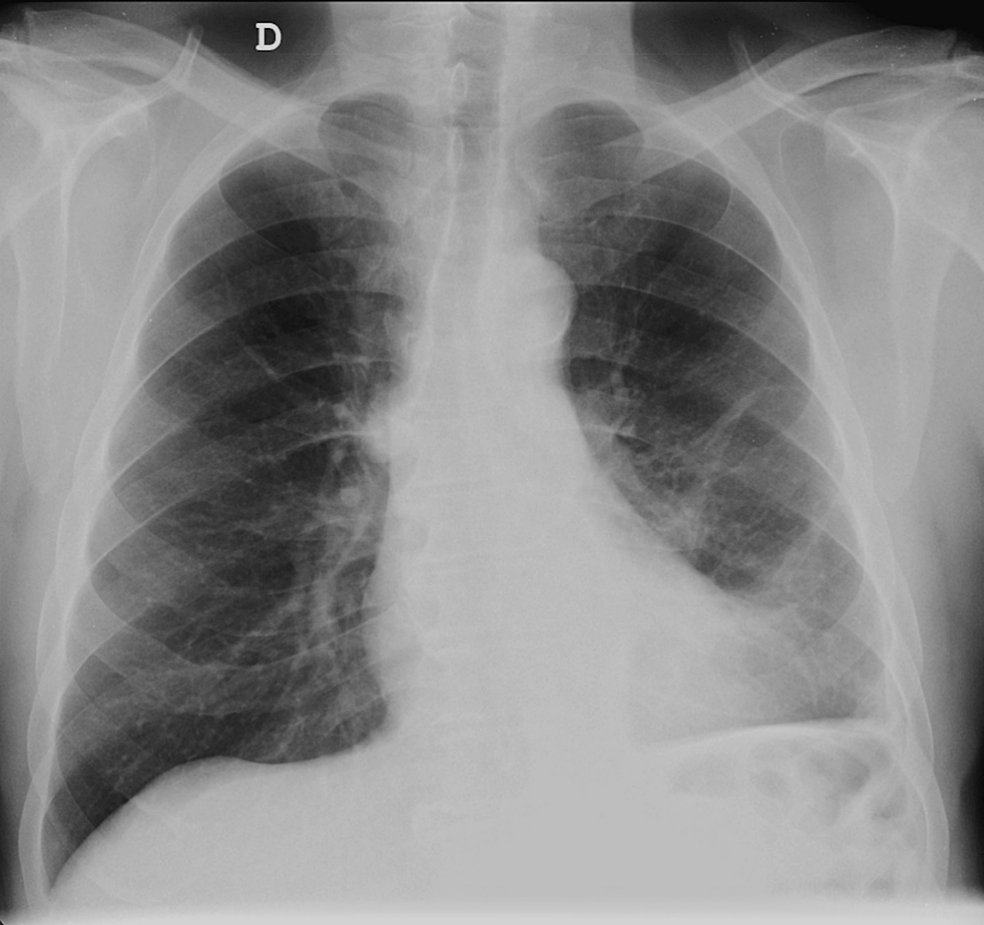
Chest X-ray three months after the episode.

The General Surgery follow-up appointment took place two months after discharge (e.g., three months after hospital admission). The patient was in good general condition, with no respiratory and/or gastrointestinal complaints. He had returned to the activities of daily living, without limitations. The control chest X-ray was normal (Figure 6).

## Discussion

Boerhaave syndrome was first described in 1724 by Hermann Boerhaave, who presented the case of a man with vomiting and chest pain after a heavy meal, and died within the first 24 hours [4]. This condition is caused by situations that suddenly increase intraesophageal pressure, such as vomiting or other strenuous efforts. It typically results in transmural and longitudinal perforation of the esophagus [1,4]. Although the perforation can occur at any location in the esophagus, it most commonly affects the posterior, lateral, and distal third of the esophagus [1,4].

This condition affects middle-aged males more frequently. Excessive alcohol consumption is the main known risk factor, followed by overeating [4].

According to the review by Brinster et al., the observed mortality rate was 18%. They also concluded that the earlier the diagnosis and surgical intervention - within 24 hours - the better the outcomes (14% vs 27% and 4% vs 14%, respectively). According to the same authors, thoracic esophageal perforation is associated with higher mortality compared to cervical or intra-abdominal perforation (27% vs 6% vs 21%) [5].

Due to the high mortality rate, the earlier the diagnosis (within the first 12 to 24 hours), the better the outcomes, with a potential survival rate of 75% [4]. The fact that the patient sought medical attention at the emergency department within the first 24 hours of symptom onset allowed for a timely and successful approach.

The clinical presentation depends on various factors, such as the location and size of the perforation, as well as the duration of symptoms [1,4]. The classical presentation - Mackler's triad - observed in only about 10% of patients [2,4], is characterized by vomiting accompanied by chest pain and subcutaneous emphysema. Other signs and symptoms such as crepitus, subcutaneous emphysema, dyspnea, and sepsis may also be present [1]. Due to the presence of these features in the presented case, early diagnostic orientation was possible.

There are no guidelines for the diagnostic and therapeutic approach to Boerhaave syndrome, as it is a rare condition. Diagnosis is based on a combination of clinical history, physical examination, and chest radiography. Chest CT can assist in confirming the diagnosis and is important for assessing the extent of the pathology and complications [6]. It can be observed that our case followed the diagnostically recommended approach.

Esophageal rupture leads to serious complications, including mediastinitis and multiorgan dysfunction. Sepsis is frequent and can occur early [7]. Other frequently reported complications include persistent leakage, fistula formation, empyema, abscess, and pneumonia [5].

Scientific evidence for the optimal therapeutic approach is scarce, and recommendations are controversial. There are no comparative studies, and the existing data in the literature are based on case series and expert opinion.

According to the review by Schipper et al., out of a total of 104 cases analyzed, 16 were treated conservatively, eight underwent endoscopic treatment, and 80 underwent surgical intervention. They found that the survival rates were 75%, 100%, and 83.5%, respectively. The proposed algorithm for the approach to this syndrome was based on the time of diagnosis and the presence of sepsis. In summary, the presence of sepsis indicates surgical intervention regardless of the elapsed time. On the other hand, in the absence of signs or symptoms of sepsis, endoscopic treatment is recommended if the diagnosis is early, within 48 hours, or conservative treatment if delayed, exceeding 48 hours [8].

The therapeutic approach can be conservative medical management, endoscopic intervention, and/or surgery. Regardless of the approach, successful treatment depends on early diagnosis, hemodynamic stabilization of the patient, and timely and appropriate therapeutic management. Concurrently, the basic therapeutic principles include oral diet avoidance (with the need for parenteral or enteral nutrition through a feeding tube), broad-spectrum antibiotic therapy covering both anaerobic and aerobic gram-negative and gram-positive bacteria, and drainage of complications [1,4,5].

Conservative management can be considered in certain situations, particularly in patients who are minimally symptomatic and show no signs of sepsis, with continuous monitoring in specialized units and the availability of diagnostic tools and multidisciplinary teams 24 hours a day [1,3]. In well-selected cases, this approach can achieve a 100% survival rate [5]. Anwuzia-Iwegbu et al. reported a successful case of conservative management, as their patient exhibited a contained leak, absence of underlying esophageal pathology, and minimal symptoms or signs of sepsis [3].

Endoscopic intervention may be indicated in stable patients with early presentation, lesions smaller than 1 cm, an experienced team, and potentially in patients who are not suitable for surgery due to significant comorbidities [1,2]. Endoscopic treatments include the placement of clips, stents, or prostheses, and, as a last resort, suturing [9].

Surgical intervention should be performed as a last resort in cases of clinical deterioration, evidence of sepsis, or treatment failure [1]. The available surgical options include primary esophageal repair with or without flap, esophagectomy or esophageal exclusion, and drainage of complications. The postoperative mortality rate is high and varies depending on the location of the perforation [1,10].

According to the meta-analysis by Kollmar et al., which included the analysis of patients who underwent surgical intervention, 227 patients were evaluated comparing primary defect repair versus esophagectomy or esophageal exclusion. They found no significant differences in mortality between the two groups. They also concluded that surgical intervention is often necessary due to the poor blood supply of the esophagus and the diffuse spillage of esophageal contents into the mediastinum [10].

In the presented case, several favorable factors, such as early clinical presentation, patient's clinical stability, absence of sepsis, and subcentimeter lesion, allowed for the non-surgical approach presented by the authors. In addition to supportive therapy, thoracic drainage procedures and endoscopic interventions involving clip placement and prosthetic placement led to successful treatment.

Conservative management endeavors to facilitate spontaneous healing of the esophageal perforation; however, there exists a potential for inadequate self-healing of the perforation. Patients subjected to conservative management should undergo vigilant clinical surveillance to evaluate for indications of clinical deterioration or the emergence of complications. A multidisciplinary approach guarantees holistic patient care, expedites timely decision-making, and enables immediate intervention in the event of complications.

## Conclusions

Early suspicion and diagnosis of this condition were crucial, allowing for immediate treatment. Emergency department physicians should consider Boerhaave syndrome in patients with acute chest pain, dyspnea, or pneumomediastinum, emphasizing the importance of suspicion, thorough medical history, focused examination and appropriate diagnostics. The multidisciplinary approach involving General Surgery, Gastroenterology, Internal Medicine, Intensive Care Medicine, Pulmonology, and Radiology facilitated the early stabilization of the patient.

The patient's stable clinical condition contributed to the successful non-surgical conservative treatment. Therapeutic options should always be individualized and based on the available resources. More case reports with varying approaches, both conservative and surgical, are needed to enhance and enrich the existing body of knowledge on the management of this condition. These case reports would contribute to a better understanding of the pathology, provide insights into successful treatment strategies, and shed light on cases where outcomes are less favorable.
